# Pancreatic Disorders in Children with Inflammatory Bowel Disease

**DOI:** 10.3390/medicina57050473

**Published:** 2021-05-11

**Authors:** Piotr Jakimiec, Katarzyna Zdanowicz, Kamila Kwiatek-Sredzinska, Aleksandra Filimoniuk, Dariusz Lebensztejn, Urszula Daniluk

**Affiliations:** Department of Pediatrics, Gastroenterology, Hepatology, Nutrition and Allergology, Medical University of Bialystok, 15-274 Bialystok, Poland; piotrjakimiec89@gmail.com (P.J.); kazdanowicz@gmail.com (K.Z.); kamila.kwiatek90@gmail.com (K.K.-S.); afilimoniuk@op.pl (A.F.); dariusz.lebensztejn@umb.edu.pl (D.L.)

**Keywords:** inflammatory bowel diseases, pancreatic diseases, extraintestinal manifestations, children

## Abstract

*Background and Objectives:* Inflammatory bowel disease (IBD) is a chronic condition and mainly affects the intestines, however, the involvement of the other organs of the gastrointestinal tract (upper part, pancreas, and liver) have been observed. The coexistence of IBD with pancreatic pathology is rare, however, it has been diagnosed more frequently during recent years in the pediatric population. This article reviews the current literature on the most common pancreatic diseases associated with IBD in the pediatric population and their relationship with IBD activity and treatment. *Materials and Methods:* We performed a systematic review of data from published studies on pancreatic disorders, also reported as extraintestinal manifestations (EIMs), among children with IBD. We searched PubMed and Web of Science to identify eligible studies published prior to 25 April 2020. *Results:* Forty-four papers were chosen for analysis after a detailed inspection, which aimed to keep only the research studies (case control studies and cohort studies) or case reports on children and only those which were written in English. The manifestations of IBD-associated pancreatic disorders range from asymptomatic increase in pancreatic enzymes activity to severe disease such as acute pancreatitis. Acute pancreatitis (AP) induced by drugs, mainly thiopurine, seems to be the most- often-reported pancreatic disease associated with IBD in children. AP associated with other than drug etiologies, and chronic pancreatitis (CP), are rarely observed in the course of pediatric IBD. The pancreatic involvement can be strictly related to the activity of IBD and can also precede the diagnosis of IBD in some pediatric patients. The course of AP is mild in most cases and may occasionally lead to the development of CP, mainly in cases with a genetic predisposition. *Conclusions:* The involvement of the pancreas in the course of IBD may be considered as an EIM or a separate co-morbid disease, but it can also be a side effect of IBD therapy, therefore a differential diagnosis should always be performed. As the number of IBD incidences with concomitant pancreatic diseases is constantly increasing in the pediatric population, it is important to include pancreatic enzymes level measurement in the workup of IBD.

## 1. Introduction

Inflammatory bowel disease (IBD) which includes Crohn’s disease (CD), ulcerative colitis (UC), and unclassified IBD, manifests as a chronic intestine disorder with not-fully-explained etiology, with periods of exacerbation and remission. The course of IBD can be complicated by inflammatory diseases which affect other organs/systems and are named as the extraintestinal manifestations (EIMs) of IBD. In a study conducted by Jose et al. from the large multicenter pediatric IBD registry (the PediIBD Consortium Registry) including 1649 patients, EIMs were reported in 6% of children [1]. Similar results were obtained by Dotson et al., who observed EIMs in 285 of 1009 enrolled children (28.2%) [2].

Pancreatic diseases are rare (0.7–1.6%) in the pediatric population and can be classified into acute pancreatitis (AP), acute recurrent pancreatitis (ARP), and chronic pancreatitis (CP), based on recent consensuses [3,4]. A diagnosis of AP is achieved by meeting at least two of the following three criteria: (1) clinical, including abdominal pain or vomiting; (2) biochemical, such as serum lipase or amylase level at least three times higher than the upper limit of normal; and (3) imaging, typical for AP [3,4]. The etiological factors of AP in children include biliary and pancreatic abnormalities, medication-associated factors, presence of underlying systemic disease, trauma, genetic predisposition, infection, metabolic disorders, and autoimmune pancreatitis (AIP). The majority of children with AP manifest mild symptoms and outcomes. Currently, there is no specific clinical and biochemical system to predict the severity of pediatric AP. Previously, it was suggested that serum lipase levels greater than or equal to seven times the upper limit of normal in the first 24 h of disease predicted the development of severe AP, however this scoring system showed low sensitivity and specificity in the recent validation reports [4].

Chronic pancreatitis in children is defined as a progressive inflammatory process that leads to destruction of pancreatic tissue and pancreatic dysfunction. The main risk factors for development of pediatric CP include genetic alterations, obstructive factors (pancreas divisum, gallstones, pancreatobiliary malunion, biliary cyst, sphincter of Oddi dysfunction, or annular pancreas), autoimmune, and toxic/metabolic factors [3,4].

In recent decades, the coexistence of hyperamylasemia/hyperlipasemia with IBD in children has been more frequently observed, possibly due to the increased incidence of IBD in children [1,5,6]. The IBD-related pancreatic disorders, reported in the literature, are a heterogeneous group of diseases/symptoms, ranging from asymptomatic increases in pancreatic enzyme activity to severe disease such as acute pancreatitis. It is unclear, whether pancreatic involvement in IBD can be considered an EIM or if it is a side effect of IBD therapy.

This article reviews the current literature on the most common pathologies of the pancreas associated with IBD in children and adolescents and their relationship with IBD activity and treatment.

## 2. Materials and Methods

**Figure 1:**
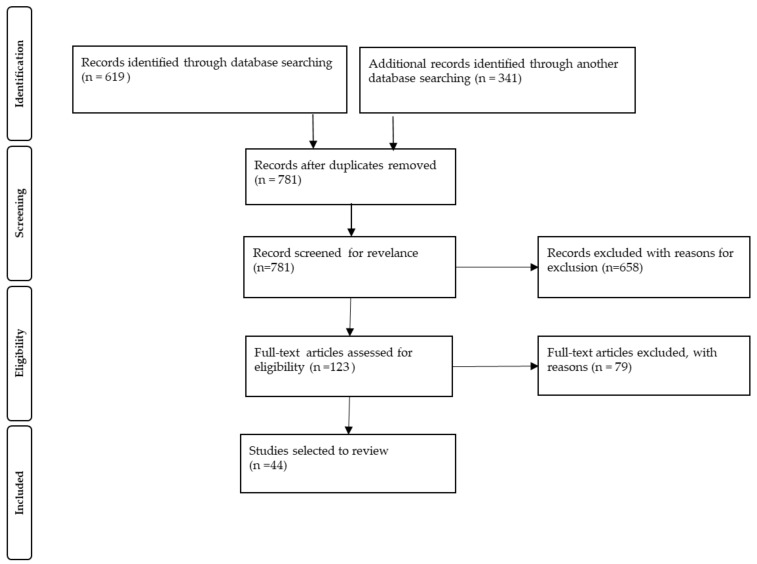
Preferred reporting items for systematic reviews and meta-analyses (PRISMA) flow diagram for study selection. Adapted from Moher et al. [7].

The PRISMA guidelines were followed [7]. PubMed (including MEDLINE) and Web of Science were searched for articles from 1 January 1986 to April 2021 using the phrases “inflammatory bowel disease” (including ulcerative colitis and Crohn’s disease) and “children” or “pediatric” in combination with “pancreatic” (to include all pancreatic disorders) or “extraintestinal manifestation”. In total, 781 records were identified in the searched electronic databases. The publications were limited to those published in the past 35 years. We excluded from the analysis abstracts from conferences, letters to the editor, meta-analyses, reviews, science papers, articles selected twice, guidelines, papers not related to pancreatic diseases in IBD, papers on extraintestinal manifestations other than pancreatic diseases in IBD, papers not related to IBD, and papers without full text available. Screening of the titles and abstracts was independently performed by three investigators. Next, the selected papers were discussed with all authors. After a detailed inspection of full-text articles, which aimed to keep the research studies (case control studies and cohort studies) or case reports only related to children and written in English, 44 papers remained. A schematic flow diagram for the selection of the studies included in this review is presented in Figure 1.

## 3. Results

In the review, we included 16 research studies shown in Table 1, and 28 case reports shown in Table 2.

The analyzed research studies were related to the pediatric population, but four studies evaluated the IBD group including adults [8,9,10,13]. One study evaluated the group of children with drug-induced complications and among them were children with IBD [19]. Another two papers are related to EIMs in IBD including pancreatic manifestation and one study on children with AIP reported IBD among the study group [1,2,20].

The results from four studies on children with IBD, including one case report, did not distinguish the type of IBD (CD or UC) in the study group [1,13,14,33]. Not all analyzed studies contained data on the type of pancreatic disease, age of patients at the diagnosis of pancreatic disease, activity of pancreatic disease, or other risk factors for the development of pancreatic disease and indicators of IBD activity. Based on the pancreatic disorders reported in the studies, three subgroup analyses were performed as follows: (1) studies on acute pancreatitis, (2) studies on chronic pancreatitis, and (3) studies related to asymptomatic elevation of pancreatic enzymes. Three articles presented data on pancreatitis without specifying whether it was acute or chronic [1,21,45]. No cases of severe disability or mortality due to pancreatitis were reported in the pediatric IBD population.

### 3.1. Studies on Acute Pancreatitis (AP)

In a study by Ghersin et al., IBD was associated with increased risk for non-immune-mediated pancreatitis in Jewish adolescents [21]. In a study by Jose et al. pancreatitis was noted in 9.6% of pediatric patients with IBD; unfortunately the type of IBD was not specified [1]. Distinct results were presented by other authors, who found pancreatitis in 0.7–2% of children with CD and in 1.3–1.4% of UC cases [2,8]. AP preceded the IBD diagnosis in 2.17% of children according to a multicenter analysis from Israel with the median lag time period of 24 (range 1–156) weeks between the episode of AP and the diagnosis of IBD [10]. Female sex was mentioned among the risk factors for AP development in children, whereas no association with IBD type, ongoing treatment, or extension of disease was found by Martinelli et al. [8]. Interestingly, the majority of children with pancreatic involvement, whether diagnosed with hyperamylasemia/hyperlipasemia or AP, presented an active IBD in this study [8]. Patients with recurrent episodes of AP and hyperamylasemia/hyperlipasemia also demonstrated higher disease activity scores at the 6- and 12-month follow-ups. The AP in all children was scored as a mild disease, and 91% of them presented normal pancreatic enzymes at 12 months follow-up [8].

The etiology of AP in IBD is heterogeneous and mainly includes the toxicity of drugs used in the treatment of IBD or a concomitant biliary lithiasis [5]. Thiopurines, like azathioprine (AZA), and their active metabolites, 6-thioguanine and 6-mercaptopurine (6-MP), belong to drugs with the highest risk for AP induction. Based on the analyzed literature, acute pancreatitis induced by a drug used to treat IBD in the pediatric population, was most frequently reported (24 from 44 studies—54%). In an analysis by Bai et al. of children hospitalized for drug-related pancreatitis, 10.9% of cases were CD and 9.1% were UC. There was no significant difference in demographic data and clinical manifestation of AP between drug-associated and non-drug-associated groups [19]. Mesalamine, steroids, and AZA were the drugs most commonly responsible for pancreatitis in these patients [19]. In the cohort study by Wintzell et al., which was based on nationwide register data from Sweden and Denmark, 1–2% of children with IBD developed AP within 3 months of initiating AZA therapy, however most events occurred within the first month. Among IBD types, no difference in frequency of affected children was detected [11]. In another report, among 72 pediatric IBD patients (57 CD and 15 UC cases) treated with AZA, four children had to stop therapy due to pancreatitis; unfortunately, the type of IBD of these patients has not been specified [14]. Additionally, only 1 in 35 Japanese children with UC developed thiopurine-induced pancreatitis which resulted in discontinuation of such therapy [15]. However, the successful introduction of 6-MP, the active metabolite of AZA, into therapy of children with CD who previously developed AZA-induced AP, has also been reported in few cases [13,18,22,23,24]. In another study, 6-MP was responsible for pancreatitis in four adolescents with CD [16]. No correlation between 6-MP dose or metabolite levels or thiopurine methyltransferase (TPMT) genotype and pancreatic toxicity was observed in the analyzed studies [12,13,14,22,25]. TMPT is an enzyme that catalyzes the methylation of 6MP and exhibits individual variability, and its low activity can lead to drug toxicity. In all reported cases of AZA/6-MP-induced AP, the symptoms of AP resolved after discontinuation of AZA/6-MP treatment [17,18,22,23,24,25]. Only isolated reports of 5-ASA-induced mild AP in children with IBD can be found in the literature [18,26,27,28,29,30,31]. Furthermore, there were three cases of AP (one mild and two severe) due to total parenteral nutrition supplemented with 20% of Intralipid combined with steroid in children with IBD as well as one case of AP due to vedolizumab, a monoclonal antibody anti-integrin α4β7 used to treat a child with UC [32,33,34]. Regarding biliary tract disease as a cause of AP in pediatric patients with IBD, only one report of biliary pancreatitis in three children is available in the literature [18]. A few cases of idiopathic pancreatitis have also been published, some preceding the diagnosis of CD [18,39,40,41].

Among other rare causes of AP found in the literature, there are reports of indigo-naturalis-induced AP in an 11-year-old boy with refractory CD, or hemorrhagic necrotizing pancreatitis in a 6-year-old girl with CD and a history of parathyroidectomy due to familial hyperparathyroidism [46,47]. An interesting case of a child with comorbidities such as CD, primary sclerosing cholangitis, and pancreatitis that recurred despite chronic use of combined anti-inflammatory and immunosuppressive therapy was described by Silbermintz et al. [45]. Only treatment with infliximab, when granulomatous pneumonia additionally occurred, resulted in improvement of lung lesions and normalization of pancreatic enzymes [45].

An association between autoimmune pancreatitis (AIP), a rare systemic disease and IBD has been documented in adult patients [5]. Among 18 children with AIP, selected from a multicenter database (INSPIRE) and from Cliniques Universitaires St-Luc (CUSL) registry (mean age at diagnosis 13 years, range 2–17), one was diagnosed with coexisting CD and three with UC [20]. Based on a literature review, the same authors identified 30 children with AIP, one of whom had CD while four had UC [20]. The case reports included in this analysis presented two adolescents with AIP and UC and one adolescent with AIP preceding the CD diagnosis. [35,37,38]. Cousin et al. also noted the coexistence of severe AIP with rectocolitis, severe liver involvement, in a 16-year-old boy [36].

### 3.2. Studies on Chronic Pancreatitis (CP)

Chronic pancreatitis (CP) is a rare condition in the pediatric population and is mainly associated with genetic alterations [4]. Only four cases of CP coexisting with IBD in children after recurrent episodes of AP have been well documented [42,43,44]. Two of these patients had the mutation in the CFTR (cystic fibrosis transmembrane conductance regulator) gene, which was possibly involved in CP development [8,42]. In the remaining two patients, the development of CP was preceded by the diagnosis of CD, including one patient who first developed idiopathic pancreatic fibrosis (IFP), a rare form of chronic pancreatitis, and one case of CP with biliary obstruction [43,44].

### 3.3. Studies Related to Asymptomatic Pancreatic Hyperenzynemia

There were only few cases of elevated pancreatic enzymes without any other symptoms of pancreatic disease in IBD children in the literature published in English [8,48,49]. Based on Martinelli study, 81.2% of patients with hyperamylasemia/hyperlipasemia (HA/HL) manifested active IBD as well as AP. After 6 months follow up, 25% of children developed AP. However, after 12 months follow up 68.7% of patients reached a complete remission in pancreatic involvement. In one case report, HL correlated with severity of UC and lipase activity decreased when remission of UC was achieved [49]. In other case report, elevated amylase activity was diagnosed as macroamylasemia [48].

## 4. Discussion

The pathogenesis of IBD-related pancreatic disorders is not fully explained. Interestingly some autoantibodies against glycoproteins of the pancreatic acini (PABs) were found in 27% to 39% of patients with CD and in 0% to 5% of patients with UC [50]. These autoantibodies are directed against CUZD1 (the CUB and zona-pellucida-like domains-containing protein 1) and GP2 (glycoprotein 2), proteins almost exclusively expressed in the exocrine pancreas. These autoantibodies are associated with perianal disease, EIMs (arthritis, dermatological, and ocular manifestations), and ASCA positivity, whereas the association with stricturing disease is incoherent. PABs have been demonstrated to be useful for the serological diagnosis of IBD, especially regarding the differentiation between CD and UC [50]. Recently published results from an animal model have revealed new findings regarding factors, such as glycoprotein 2 (GP2), related to the pancreatic-colon axis. The GP2, secreted by pancreatic cells, is expressed on M cells located in the small intestine and initiates antigen-specific mucosal immune responses that control bacterial invasion into intestinal epithelial cells. In a chemically induced colitis in mice, an increase of GP2 expression in the pancreas has been observed, which has been an effect of enhanced production of pro-inflammatory cytokines such as TNF [51]. The significant increase of luminal GP2 level was also reported in patients with CD. It is possible that by disrupting the mucosal barrier by inflammation, different species of bacteria bound to GP2 may translocate and then induce the production of GP2 autoantibodies in colitis. Nevertheless, the role the autoantibodies in the pathogenesis of pancreatic diseases in IBD is still unknown. Interestingly, elevated serum GP2 levels were noted in patients with pancreatitis and in CD patients [51,52]. Involvement of the pancreas in IBD may be connected with GP2 dysfunction or an increased level of anti-GP2 autoantibodies. However, more research is needed to evaluate the pancreatic–colon axis. Recent studies on metabolomics, lipidomics, and proteomics in IBD and pancreatic diseases might help to find the common pathway of both diseases [53,54].

AP is the most common pancreatic disease associated with IBD [5]. In fact, IBD is listed among the five most important causes of AP in children [19,55]. The incidence rate of AP in children with IBD seems to be higher than in adults [1,6,9,56,57,58]. Based on the studies the pancreatic involvement could be strictly related to the activity of IBD in some pediatric patients [6,8]. In active IBD, intestinal inflammation combined with increased permeability of intestinal wall may cause the pathological reabsorption of pancreatic enzymes resulting in the elevation of serum pancreatic enzymes. On the other hand, the fecal calprotectin, the known marker of IBD, was reported to be also increased in pancreatitis, but not as high as in IBD [59,60]. The etiology of AP in the IBD course is heterogeneous and mainly includes a biliary lithiasis concomitant with IBD or the toxicity of drugs used in the treatment of IBD [5]. Most drugs used to treat IBD may be responsible for the development of AP. However, it is difficult to determine whether the pancreatitis is an extraintestinal manifestation of IBD or it is induced by drugs used in IBD therapy, especially at the onset of the IBD management [2,49]. In the pediatric population, the course of acute pancreatitis in most cases is mild, regardless of whether it is drug- induced or not [19]. The potential mechanisms of drug-induced pancreatitis are presented in Table 3.

Drug-induced pancreatitis usually arises in the first weeks of exposure and presents in a mild form with normalization after the discontinuation of the therapy [17,18,22,23,24,25,26,27,28,30,31,57]. In practice, reintroduction of the drug is not a standard procedure [55]. Thiopurines, 5-aminosalicylates (5-ASA), and steroids may induce direct toxicity, hypersensitivity, dyslipidemia, and secondary hypercalcemia [55]. Thiopurines, like AZA, and their active metabolites, 6-thioguanine (6-TG) and 6-MP, belong to drugs with the highest risk for AP induction. The pathogenesis of AZA-induced AP is not clear; an immune-mediated mechanism, a hypersensitivity to the drug, or idiosyncratic adverse effect have been postulated [24,62,63,64,65,66]. The association of AZA-induced AP with TMPT mutation was not confirmed in IBD patient [12,13,14,22,25,66]. The current guidelines for management in pediatric IBD suggest the discontinuation of thiopurine in the case of drug-associated AP, however, it is recommended to differentiate true thiopurine-related toxicity from extraintestinal manifestation of IBD reflected as pancreatitis. The incidence of AP induced by 5-ASA in the pediatric population appears to be very low as it is mainly presented in case reports [18,26,27,28,29,30,31]. It can occur despite any other mode of administration, such as oral or rectal administration. In the study on adults, the incidence of mesalamine-induced pancreatitis was estimated at one/million days of treatment with most reported cases of AP occurring within the first 6 weeks of therapy in a non-dose-related fashion and with improvement a few days after drug discontinuation [68]. In addition, it was recently reported that vedolizumab, a monoclonal antibody used in a new treatment option for IBD, was responsible for the development of pancreatitis in an unexplained manner, suggesting a dysregulation of the immune response [32]. Another risk factor for AP, biliary lithiasis, is approximately twice as common in adults with CD as in general population [5]. The increased prevalence of cholelithiasis in CD is determined by the extension and the location of inflammatory lesions in the small bowel and the extension of surgical resections of the small intestine [65]. However, when considering pediatric patients, there is only one report regarding biliary disease as a cause of AP in three children with IBD [18]. Isolated reports of other rare causes of AP in children with IBD show that there are many different and even unexpected triggers for pancreatitis in this pediatric population [33,34,46].

AIP is a rare disorder of presumed autoimmune etiology that is classified into two types. Type 1 is the most common form and the pancreas is involved as a part of a systemic IgG4-related disease. Type 2 AIP, on the contrary, is not characterized by elevated IgG4 levels, affects younger patients, and is frequently associated with IBD [5]. The prevalence of IBD in patients with AIP is higher than in the general population and in most cases UC is diagnosed [20,35,36,37,38,55].

IBD-associated CP is an extremely rare condition in children [55]. Based on the reported cases in children, the development of CP often preceded the diagnosis of IBD [42,43,44]. Interestingly, both conditions, despite the lack of a documented cause-and-effect relationship, share common features such as inflammation and fibrosis. In adults, idiopathic CP in IBD is reported in 1.2–1.5% of the cases [5].

Hyperamylasemia/hyperlipasemia (HA/HL) may preceded AP development in children with IBD [8]. In some cases, it may occur without any significant reason as an idiopathic form or EIMs of IBD [44,45]. In adult patients with IBD, an asymptomatic HA or HL was reported in 16–16.7% of patients with CD and 11.4–21% of patients with UC, without morphological abnormalities on ultrasound and with no association with the disease activity, duration, or medication [69,70]. Similar results were obtained in children with IBD in the study by Stawarski et al., in which the benign HA was observed in 27.3% of patients with CD and 12.7% of cases with UC, but HL was noted only in 3.8% children with UC [6]. In the differential diagnosis, it is important to remember about macroenzymes, the high-molecular-mass complexes in serum. Incorrect interpretation of the result of serum pancreatic enzymes caused by macroenzymes may lead to unnecessary therapy and a wrong diagnosis.

## 5. Conclusions

The manifestations of pancreatic pathologies associated with IBD range from an asymptomatic increase in the activity of pancreatic enzymes to severe disorders such as acute pancreatitis. AP induced by a drug, mainly thiopurine, seems to be the most-often-reported pancreatic disease associated with IBD in children. AP associated with other than drug etiologies and CP, is rarely observed in the course of pediatric IBD. The pancreatic involvement can be strictly related to the activity of IBD and can also precede the diagnosis of IBD in some pediatric patients. The course of AP is mild in most cases and may occasionally lead to the development of CP, mainly in cases with genetic predisposition. The involvement of the pancreas in the course of IBD may be considered an EIM or a separate co-morbid disease, but it can also be a side effect of IBD therapy; therefore a differential diagnosis should always be performed. As the number of IBD incidences with concomitant pancreatic diseases is constantly increasing in the pediatric population, it is important to include pancreatic enzyme level measurements in the workup of IBD.

## Figures and Tables

**Table 1:** Pancreatic disorders reported in children with IBD based on research studies.

Authors, Year of the Publication	Pancreatic Disorder	Number of Affected Children with IBD			Etiology of Pancreatic Disease	Severity of Pancreatic Disease	Comments Analyzed Population; Mean Age (Ranges) at the Time of Pancreatic Involvement
		CD *n* (%)	UC *n* (%)	IBD-U			
Martinelli et al., 2015 [8]	AP HA/HL	6/284 (2%) 7/284 (2%)	4/290 (1.4%) 8/290 (2.8%)	1/48 (2%) 1/48 (2%)	No data	Mild	IBD with pancreatic involvement; age 12.3 (5.4–25.9); active IBD in 85.1% of both AP and HA/HL group; in 18.5% of cases pancreatic involvement at the time of IBD diagnosis
Weber et al., 1993 [9]	AP	1/12 (8.3%) 1/12 (8.3%)	NA	NA	Sulfasalazine Pancreas divisum	No data	CD; age of study group: 10–50 yr; only two children (10 yr and 18 yr) in the study group; 10-yr-old boy developed AP induced by sulfasalazine at the time of CD diagnosis, symptoms resolved after drug discontinuation 18-yr-old girl developed AP one year after CD, AP due to pancreas divisum
Broide et al., 2011 [10]	AP	6/460 (1.3%)	4/460 (0.9%)	No data	Idiopathic	Mild	AP preceded the IBD diagnosis in 12/460 patients including two adults; mean age of children was 13 ± 4.8 (range 3–19) yr, the description of the study group includes adults; nine patients had moderate to severe IBD
Wintzell et al., 2019 [11]	AP	21/1923 (1.1%)	19/1451 (1.3%)	No data	AZA	No data	IBD treated with AZA; age at the time of pancreatic involvement—no data; similar rate of AP in boys and girls and UC and CD
Dubinsky et al., 2000 [12]	AP HA/HL	1/92 (1%) children with IBD6/92 (6.5%) children with IBD			AZA/6MP	No data	IBD treated with AZA/6MP; age at the time of pancreatic involvement—no data; IBD type - no data; normal TMPT genotype, no correlation between 6-MP dose or metabolite levels and pancreatic toxicity
Hindorf et al., 2006 [13]	AP	2/79 (2.5%) children with IBD		No data	AZA	No data	IBD treated with AZA; age of the study group: 17–51 yr; age at the time of pancreatic involvement—no data; there was no difference in TPMT activity between patients with pancreatitis and patients without adverse event
De Ridder et al., 2006 [14]	AP	4/72 (5.6%) children with IBD		No data	AZA	No data	IBD treated with AZA; age at the time of pancreatic involvement—no data; IBD type not specified; normal TMPT genotype
Tajiri et al., 2008 [15]	AP	NA	1/35	No data	AZA/6MP	No data	UC treated with AZA/6MP; age at the time of pancreatic involvement—no data.
Cuffari et al., 1996 [16]	AP	4/15 (16%)	NA	No data	6MP	No data	CD treated with 6MP; age at the time of pancreatic involvement—no data
Kirschner et al., 1998 [17]	AP	2/95 (2%)	2/95 (2%)	0/95	AZA/6MP	No data	IBD treated with AZA/6MP; age 11.5–16.2 yr; drug discontinued
Keljo et al., 1997 [18]	AP AP AP AP	1/10 2/10 2/10 2/10	0/10 0/10 1/10 2/10	No data	5-ASA AZA Biliary pancreatitis Idiopathic	No data No data No data Mild	8.6-yr-old girl, AP symptoms resolved after 5-ASA discontinuation 17.9-yr-old girl; 12.3-yr-old girl; in both cases symptoms resolved 17.2-yr-old girl with CD; 13.7-yr-old girl with CD; 12.8-yr-old girl with UC 12.7-yr-old boy and 10.8-yr-old girl with CD; 9.9-yr-old and 14.9-yr-old girls with UC.
Bai et al., 2011 [19]	AP	6/51 (10.9%)	5/51 (9.1%)	No data	Drug	No data	Drug-induced pancreatitis; no demographic data on patients with CD and UC
Scheers et al., 2017 [20]	AIP	1/16 (6.3%)	3/16 (18.8%)	No data	AIP	No data	AIP; no demographic data on patients with CD and UC
Jose et.al., 2009 [1]	Pancreatitis	37/387 (9.6%) children with IBD		No data	No data	No data	IBD and EIMs; age at the time of pancreatic involvement—no data; IBD type not specified, pancreatitis type not specified
Dotson et al., 2010 [2]	Pancreatitis	5/728 (0.7%)	4/281 (1.4%)	No data	No data	No data	IBD and EIMs; age at the time of pancreatic involvement—no data; pancreatitis type not specified
Ghersin et al., 2020 [21]	Pancreatitis	5/231 (0.8%)	3/231 (1%)	No data	No data	No data	Jewish adolescents with IBD; age at the time of pancreatic involvement—no data; IBD type—no data, pancreatitis type—no data

NA, not available; IBD, inflammatory bowel disease; CD, Crohn’s disease; UC, ulcerative colitis; AP, acute pancreatitis; IBD-U, IBD unclassified; CP, chronic pancreatitis; AZA, azathioprine; 6MP, 6-mercaptopurine; 5-ASA, 5-aminosalicylic acid; EIMs, extraintestinal manifestations; AIP, autoimmune pancreatitis; TMPT, thiopurine methyltransferase; HL, hyperlipasemia; HA, hyperamylasemia; *n*, number of patients.

**Table 2:** Pancreatic disorders reported in children with IBD based on case reports.

Authors, Year of the Publication	Pancreatic Disorder; AP Severity	Etiology of Pancreatic Disease	CD *n* (%)	UC *n* (%)	Comment
Gallego-Gutierrez et al., 2015 [22]	AP; mild, moderate	AZA	2 cases (10- and 13-yr-old)	NA	AP symptoms resolved after AZA discontinuation; normal TMPT genotype
Yi et al., 2012 [23]	AP; severity-no data	AZA/6MP	1 case (14-yr-old)	NA	AP symptoms resolved after AZA/6MP discontinuation
Ledder et al., 2013 [24]	AP; mild	AZA	4 cases (11-, 13-, 13-, 14-yr-old)	NA	AP symptoms resolved after AZA discontinuation; 6MP was successfully used
Mishra et al., 2020 [25]	AP; mild	AZA	1 case (16-yr-old)	NA	AP symptoms resolved after AZA discontinuation; normal TMPT genotype
Abdullah et al., 1993 [26]	AP; severity-no data	ASA	NA	1 case (12-yr-old)	Sulfasalazine/mesalamine-induced AP; AP symptoms resolved after drug discontinuation
Paul et al., 2000 [27]	AP; severity-no data	ASA	NA	1 case (10-yr-old)	AP symptoms resolved after drug discontinuation
Radke et al., 1993 [28]	AP; moderate	ASA	1 case (12-yr-old)	NA	AP symptoms resolved after drug discontinuation
Garau et al., 1994 [29]	AP; severity-no data	ASA	NA	3 cases (12, 12 and 13-yr-old)	AP symptoms resolved after drug discontinuation in all cases, but in one case intractable severe colitis unresponsive to intensive therapy led to subtotal colectomy
Paerregaard et al., 1997 [30]	AP; severity-no data	ASA	NA	1 case (7-yr-old)	AP induced by oral or rectal administration of 5-ASA; AP symptoms resolved after drug discontinuation
Chung et al., 2015 [31]	AP; severity-no data	ASA	NA	1 case (11-yr-old)	AP coexisting with pneumonitis induced by mesalazine; AP symptoms resolved after drug discontinuation
Lopez et al., 2018 [32]	AP; severity-no data	Vedolizumab	NA	1 case (14-yr-old)	AP symptoms resolved after drug discontinuation, but refractory colitis led to subtotal colectomy
Noseworthy et al., 1983 [33]	AP, severe	Intralipid-supplemented TPN	2 cases with IBD (not specified type of IBD)		AP developed after 7 weeks of 20% Intralipid-supplemented TPN combined with high dose of steroid
Lashner et al., 1986 [34]	AP, mild	Intralipid-supplemented TPN	1 case (17-yr-old)	NA	AP developed after 6 weeks of 20% Intralipid-supplemented TPN combined with steroid and oral foods (small amount)
Gouveia et al., 2018 [35]	AP; severity-no data	AIP	NA	1 case (13-yr-old)	AIP preceded UC diagnosis; AIP therapy with an endoscopic retrograde cholangiopancreatography (ERCP) with stent placement induced sustain AIP remission
Cousin et al., 2018 [36]	AP; severe	AIP type 2	NA	1 case (16-yr-old)	AP with elevated IgG4, cholestasis with cirrhosis and UC
Kolasinski et al., 2017 [37]	AP; severity-no data	AIP type 2	NA	1 case (15-yr-old)	AIP with elevated IgG4 and coexisted with UC with no intestinal complaints
Dogan et al., 2020 [38]	AP; severity-no data	AIP	1 case (16-yr-old)	NA	AIP with elevated IgG4 preceded CD diagnosis
Kugathasan at al., 2002 [39]	AP; severity-no data	Idiopathic	3 cases (12, 13 and 16-yr-old)	NA	AP preceded CD development
Endo et al., 2021 [40]	AP; severity-no data	Idiopathic	1 case (16-yr-old)	NA	AP preceded CD diagnosis
Watanabe 2008 [41]	AP, mild	idiopathic	NA	1 case (15-yr-old)	AP coexisted with parotitis
Knafelz et al., 2013 [42]	CP	CFTR mutation	1 case (4-yr-old)	NA	CP preceded CD development
Evans et al., 1996 [43]	CP	Biliary tract obstruction	1 case (13-yr-old)	NA	CP with biliary tract obstruction preceded CD development
Potamianos et al., 2000 [44]	CP	Idiopathic	1 case (16-yr-old)	NA	Fibrosing pancreatitis preceded CD development
Silbermintz et al., 2006 [45]	Pancreatitis	Idiopathic	1 case (10-yr-old)	NA	Coexistence of CD, granulomatous pneumonitis, and PCS
Kim et al., 2019 [46]	AP; severity-no data	Indigo-naturalis	1 case (11-yr-old)	NA	Boy with severe CD
Briem-Richter et al., 2010 [47]	AP; severity-no data	Hemorrhagic necrotizing pancreatitis	1 case (6-yr-old)	NA	CD and familial hyperparathyreoidism
Venkataraman et al., 2012 [48]	HA	Idiopathic	1 case (13-yr-old)	NA	CD and macroamylasemia
Ray et al., 2016 [49]	HL	Idiopathic	NA	1 case (13-yr-old)	HL correlated with severity of UC and lipase activity decreased when remission of UC was achieved

NA, not available; IBD, inflammatory bowel disease; CD, Crohn’s disease; UC, ulcerative colitis; AP, acute pancreatitis; CP, chronic pancreatitis; AZA, azathioprine; 5-ASA, 5-aminosalicylic acid; TNP, total parenteral nutrition; AIP, autoimmune pancreatitis; 6′MP, 6-mercaptopurine; HL, hyperlipasemia; HA, hyperamylasemia; *n,* number of patients.

**Table 3:** Potential mechanisms of drug-induced pancreatitis.

Drug	Potential Mechanism of Pancreatitis
5-ASA	Hypersensitivity reaction [46] Increased pancreatic duct permeability [61]
AZA	Direct toxic reaction [62] Genetic predisposition [63] Immunological reaction [24,64] Idiosyncratic reaction [24,65,66] Conflicting data: inosine triphosphate pyrophosphatase (ITPase) deficiency [14,67]
6-MP	Direct toxic reaction [62] Genetic predisposition [63]
Vedolizumab	Dysregulation of immune response [32]
Intralipid-supplemented total parental nutrition (TPN)	Hyperlipidemia in combination with high dose of steroid [33]
